# Quantification of the Landscape for Revealing the Underlying Mechanism of Intestinal-Type Gastric Cancer

**DOI:** 10.3389/fonc.2022.853768

**Published:** 2022-05-03

**Authors:** Chong Yu, Jin Wang

**Affiliations:** ^1^ Department of Statistics, Jilin University of Finance and Economics, Changchun, Jilin, China; ^2^ Department of Chemistry and of Physics and Astronomy, State University of New York at Stony Brook, Stony Brook, NY, United States

**Keywords:** gastric cancer, *Helicobacter pylori*, intestinal type, gene regulatory network, landscape, flux

## Abstract

Gastric cancer is a daunting disease with a tragic impact on global health. It is the fourth most common cancer and has become the second most frequent cause of cancer death in recent times. According to the Lauren classification, gastric cancer can be classified into two types: intestinal and diffuse. Intestinal-type gastric cancer (IGC) is more common in elderly people, and atrophic gastritis (AG) and intestinal metaplasia (IM) have been proven to be the main premalignant causes of intestinal-type gastric cancer. In turn, *Helicobacter pylori* infection has been identified as the most significant cause of AG and IM. In this study, we determine the mechanism of IGC progression and how *H. pylori* infection induces IGC. Through researching the relevant literature, we identified the key genes associated with gastric cancer and the specific genes associated with IGC. We then use hese genes to build up a gene regulatory network for IGC. Based on this gene regulatory network, we quantify the IGC landscape. Within this landscape, there are three stable states, which are classified as the normal, AG, and gastric cancer states. Through landscape topography, we can determine the biological features and progression process of IGC. To investigate the influence of *H. pylori* infection on IGC, we simulated different degrees of *H. pylori* infection. As the *H. pylori* infection becomes more serious, the landscape topography changes accordingly. A fourth state, named the intestinal metaplasia (IM) state, emerges on the landscape and is associated with a very high risk of developing gastric cancer. The emergence of this state is due to the interactions/regulations among genes. Through variations in the landscape topography, we can determine the influence of *H. pylori* infection on IGC. Finally, we use global sensitivity analysis to research the regulations most sensitive to IGC prevention or therapies. This study presents a new approach and a novel model with which to explore the mechanism of IGC. The simulations of different degrees of *H. pylori* infection can provide us with a systematic view of IGC progression. The key regulations found can give us some insight and guidance for clinical trials and experimental studies.

## 1 Introduction

Cancer has long been considered the most daunting disease, and gastric cancer is the second most aggressive cancer, having a tremendous, large-scale impact on global health. Despite a huge amount of research, gastric cancer remains the fourth most common cause of cancer-related deaths worldwide (1). Despite a decline in incidence in the last several decades, the prognosis for gastric cancer is still very poor. The five-year survival rates for gastric cancer are less than 20% (2). Early-stage gastric cancer has a better prognosis, with five-year survival rates of up to 95% (3). According to the Lauren classification, gastric cancer can be divided into two types: intestinal and diffuse. The intestinal type occurs more frequently, in about 54% of cases, and more commonly in men and elderly people (4, 5). Atrophic gastritis (AG) and intestinal metaplasia (IM) have been proven to be the main premalignant factors in the intestinal type of gastric cancer (2).

Intestinal-type gastric cancer (IGC) is caused mainly by environmental factors such as salty food, alcohol, and cigarette smoking. These factors may contribute to AG, which is considered one of the main precursor lesions of IGC (6). Moreover, *Helicobacter pylori* infection can increase the risk of IGC developed from AG. The stomach is the natural reservoir of *H. pylori*. Studies show that about 50% of the world’s human population is chronically colonized by *H. pylori* and about 15% of infected people develop gastric cancer from AG and IM (7). *H. pylori* infection may cause epithelial damage, which can trigger a multistep progression to gastric cancer from AG, gastric atrophy, and IM (8, 9). These changes are mainly caused by epigenetic alterations (10). Epigenetic modifications such as DNA methylation and histone modifications can alter cell cycles. Aberrant DNA methylation can also induce IGC formation.

However, the oncogene gene c-met is related to the development of about 20% of IGC cases, and alterations in c-met have also been associated with many types of diseases, particularly diseases of the digestive system (11). IM, dysplasia, and invasive carcinomas are associated with K-Ras mutations (12). Abnormal expressions of tumor suppressor genes, such as TP53 and APC, are found in many IGC subjects (13). Therefore, the development of IGC is genetic and epigenetic, and neither of these two factors can be ignored. In the study of tumor biology, network-based models have received more and more attention recently. Many studies have shown that molecular targeted therapy can help predict cancer biomarkers, design network-based anti-cancer therapies, and provide clinical strategies for cancer studies (14–17). This is because gene regulatory networks can help resolve key issues in cancer research by reflecting not just information at the genetic level but also epigenetic information embedded in gene regulation strengths.

In this study, we investigate IGC formation and mechanisms at both the genetic and epigenetic levels. From literature research, we built an IGC-related gene regulatory network. Some other network-based methods, such as the correlation network (18), do not contain information on gene regulations. The regulations in networks built using regression methods do not contain regulatory directions (19). Networks built using other machine learning methods, such as dynamic Bayesian networks, contain regulatory directions and feedback loops, but researchers must consider biases in the algorithm’s accuracy (20). The regulations in the gene regulatory network we build are all from the experimental literature and contain the regulation directions and regulatory types (activation or repression) from experiments that are more reliable compared to high-throughput data mining. Based on the IGC gene regulatory network, we quantify the corresponding landscape. There are three stable states on the landscape—the normal, AG, and gastric cancer (IGC) states. The landscape can give us a better understanding of IGC formation through molecular mechanisms and epigenetic information. The dominant paths between state attractors can be quantified and used to understand the development and progression of IGC.

To investigate how *H. pylori* infection can increase the risk of IGC developed from AG and IM, we simulated different degrees of *H. pylori* infection to provide a global perspective on IGC development. Finally, we use global sensitivity analysis to determine which regulations are more sensitive to IGC prevention and treatment strategies. Three regulations are found—RAS → HIF-1*α*, ZEB → TGF-*β*, and HIF-1*α*→ RAS. These results may guide clinical treatment and the design of drugs based on network strategies.

## 2 Development of Intestinal Gastric Cancer Model

**Figure 1:**
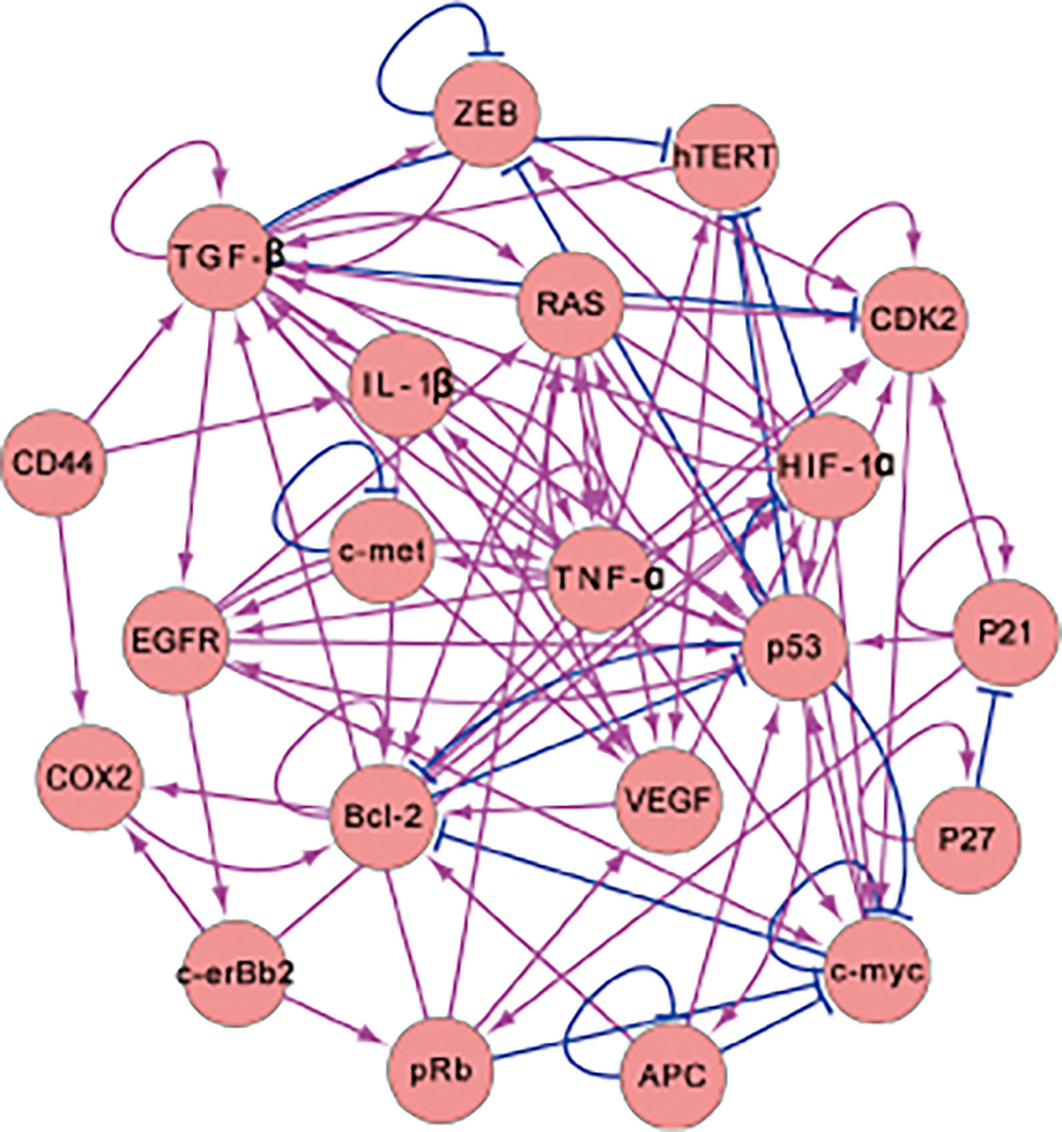
The 17-node gene regulatory network for intestinal-gastric cancer. The network contains 17 genes and 82 regulations (There are 67 activating and 15 repressing regulators. The arrows and the blunted arrows denote the activating and repressing regulations, respectively).

We researched the literature to collect information on genes related to gastric cancer and then used these gastric genes to build the gastric-cancer-related gene regulatory network shown in **Figure 1**. In **Figure 1**, there are 17 genes and 82 regulations. The activating regulations are represented by arrows, and the repression regulations are represented by blunted arrows. The 17 genes were identified by mining the gastric cancer literature. We first collected the genes that are highlighted in publications about gastric cancer, then those related to the 10 hallmarks of cancer and mentioned in the gastric cancer literature. We put these genes into the EVEX database to do text mining and search for interactions. The regulations were collected and the results with low confidence were removed. Very high or high confidence regulations were kept. Moreover, we examined the literature to make sure the regulations identified were correct. All of these results are from the experimental literature. Such literature can clarify how one gene or protein influences another gene or protein. We then identified two genes and determined the regulated relationship between them. These interactions are listed in the **Supplementary Material**. The genes Bcl-2, c-erBb2, and K-ras are the specific genes for IGC. Another 14 genes are crucial for gastric cancer. The genes DCC and Beta-caterin are also specific genes associated with IGC. However, regulation of these two genes in the gene regulatory network does not have feedback loops. Therefore, we remove them from the gene regulatory network. Feedback loops are important because they ensure interactions are non-trivial. Genes with feedback loops must be included in the network. The behavior of genes without feedback loops is relatively straightforward to understand. To simplify the computational process, we reduce the dimensionality by taking these genes as one effective node instead of many until feedback occurs. When the gene does not have incoming edges (there is no gene activating or repressing it), gene expression or protein concentration will be determined by its own self-generation and degradation. Even if a gene has no feedback edges, it can regulate downstream genes. It can influence other genes or proteins through its regulation of others, which depends on its expression or concentration. The regulation of the downstream genes can be manifested by a regulation parameter that is kept constant in the process and determined from the ODEs of the model. Due to the constant regulation, the influence of the gene on others is kept constant. Therefore, we can simplify the model by using the network without it to avoid redundant computations. We list the entire network in the **Supplementary Material**.

In the gene regulatory network, P53 and APC are vital for gastric disease (13, 21) and are both tumor suppressor genes. Abnormalities in TP53 expression have also been observed in *H. pylori*-related AG, IM, dysplasia, and others (22). C-myc participates in cell proliferation and apoptosis, which is significant for gastric cancer and other digestive-system-related cancers (23). HIF-1*α* is involved in glycolysis pathways for hypoxia (24) and is a critical prognosis element for gastric cancer (25). The RAS gene participates in certain cellular functions such as cell proliferation, differentiation, survival, and apoptosis (26). TGF-*β* plays a major role in cancer metastasis and participates in the transduction of self-sufficiency growth signals (27). TNF-*α* is involved in gastric cancer progression, such as in invasion and metastasis (28). The gene c-erbB2 encodes a type of kinase that shows a response to prognosis and is associated with IGC therapy (29). ZEB is a key gene for epithelial–mesenchymal transitions (EMT) that promotes cancer metastasis (30). EGFR is a vital prognostic factor of IGC that is related to the transduction of proliferative signals (31). The VEGF gene is often highlighted in IGC prognosis and has a vital gene response to angiogenesis (32, 33). The c-met gene is a prominent drug target of IGC (34). Bcl-2 plays a key role in apoptosis, and the dysfunction of Bcl-2 is the basis of carcinogenesis (35). COX2 is a key player in IGC development and is associated with risks for numerous types of cancer (36). hTERT is a potent part of IGC and is related to unlimited DNA replication (37). CDK2 is known as an evading growth suppressor and is indispensable in gastric cancer therapy (38). IL-1*β* is a cytokine associated with lesions, inflammation, and wound healing (39).

Once the gene regulatory network has been developed, we can use ordinary differential equations to describe the dynamics of the related network, with the equations as shown below:


$$\frac{dX_{i}}{dt}=F_{i}=g_{i}\overset{n_{i}}{\underset{j=1}{\prod }}H_{ji}-k_{i}X_{i}\;(1)$$

In Eq. (1), $\frac{dX_{i}}{dt}$ represents the gene expression (protein concentrations), which changes with respect to time. The parameters *g* and *k* are used to illustrate the protein generation rate and the protein self-degradation rate, respectively. *X_i_* is used to represent the gene expression or the amount of protein that causes the transcript of the gene *i*. The subscript *j* represents the gene regulating the gene *i*. *n_i_* is the amount of gene regulating the gene *i*. *H_ji_* quantifies the regulations among genes through a Hill function (40), which can be defined as the following:


$$H_{ji}=\frac{S_{ji}^{n}}{S_{ji}^{n}+X_{j}^{n}}+\lambda_{ji}^{r}\frac{X_{j}^{n}}{S_{ji}^{n}+X_{j}^{n}}\;(2)$$

Here, the parameter *S* denotes the threshold, which is the half-maximum value of the sigmoid function. When the value of *S* is very large, the regulation strength will tend toward a fixed value of 1. When the value of *S* is very small, the regulation strength will tend toward another fixed value, $\lambda_{ji}^{r}$ . To keep the regulation strength in an appropriate range, *S* is set to be 2.5. The meanings of the subscripts *i* and *j* are equivalent to those in Eq. (1). The parameter *n* denotes the steepness of the sigmoid function and demonstrates the protein cooperatives. In a biochemical system, a protein binding complex can be a monomer, dimer, trimer, tetramer, etc. In our system, since tetramers are more frequent, the parameter *n* is set to 4. The parameter *λ_ji_* is defined to be greater than 1, and denotes the regulation strength of *X_j_* in regulating *X_i_*. The parameter *r* denotes the regulation type (*r* = +1 represents the activation type and *r* = -1 represents the inhibition type). The parameters *g* and *k* denote the protein generation rate and the protein self-degradation rate, respectively. To simplify the calculation of the whole system, we set them to be comparable with each other, with *g* = 1 and *k* = 1.

The parameter *λ* is a matrix representing the weight of the gene regulatory network (see **Table S1** of the **Supplementary Material**). The gene regulatory network contains 17 genes. Therefore, 17 ODEs were used to describe the dynamics of the whole biological system. The weights of the network are different, and related to the gene-gene regulation strengths. We determined the weights of the network from the biological functions and gene expression in different stages of gastric cancer. For example, P53 is a tumor suppressor gene, and the gene expression of P53 will be high in the normal state and low in the gastric cancer state. Another 16 gene expressions are consistent with the literature results. If the behavior of gene expressions is inconsistent with the results in the literature, we modify the parameters of the network accordingly until the simulated gene expressions are consistent with experiments. As there are ranges of parameters that can produce similar behavior, we vary the parameters by about 10% and ensure that the gene expressions of the normal and cancer states, as well as the associated landscape topography, do not change significantly. We believe this gives a range of parameters that lead to behavior consistent with the literature.

## 3 Results and Discussion

### 3.1 The IGC Landscape and Related Kinetic Paths

**Figure 2:**
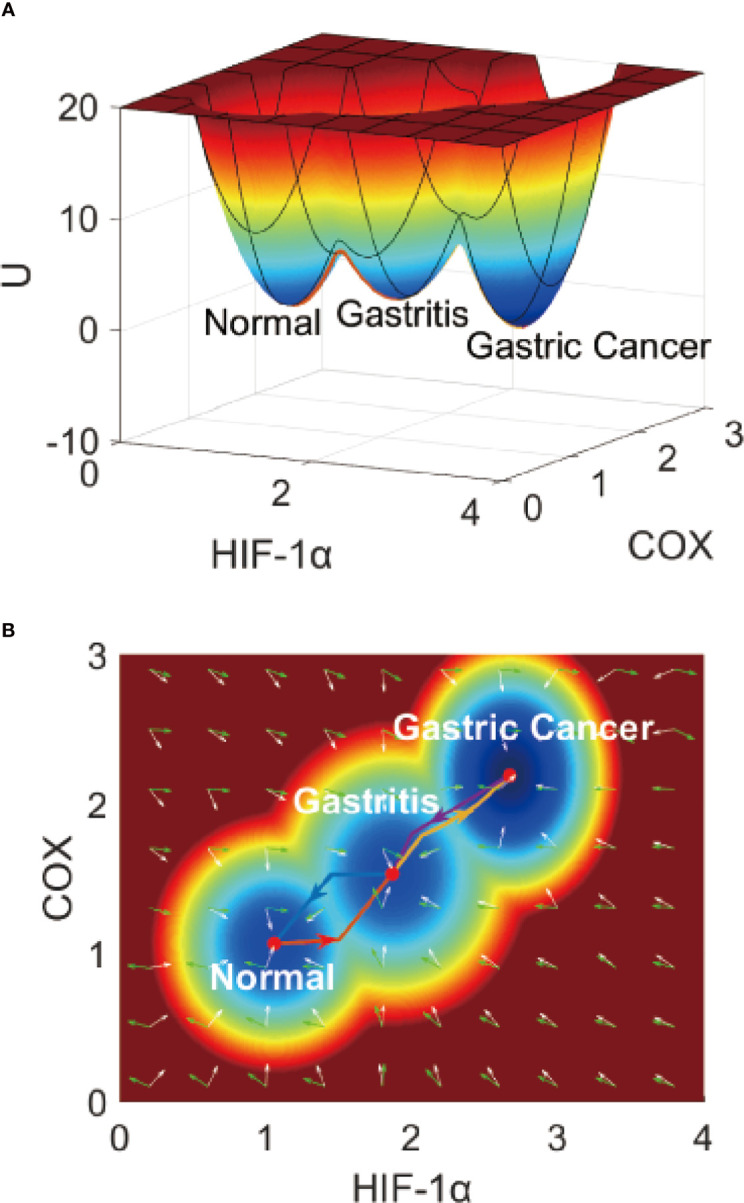
The IGC landscape, which contains three stable states. **(A)** The 3-dimensional landscape of IGC. **(B)** The 2-dimensional landscape of IGC. The lines in red, blue, yellow, and purple represent the dominant kinetic path from the normal to the AG state, from the AG to the normal state, from the AG to the gastric cancer state, and from the gastric cancer to the AG state, respectively. The white arrows denote the negative gradient of the potential landscape, and green arrows denote the curl flux force of the potential landscape.

The gene regulatory network (**Figure 1**) of IGC contains 17 genes. Through the collection of our simulation trajectories and associated statistics, we can quantify the landscape on a 17-dimensional probability distribution. The related potential landscape *U* can be defined as *U* = -*lnP_ss_* (41, 42). *P_ss_* is the probability of the steady-states, with’ss’ being an abbreviation of steady state. It is difficult to visually display a 17-dimensional landscape, so we take two genes or dimensions (*HIF* - 1*α* and COX2, two genes very crucial to IGC) to visualize the landscape clearly. In **Figure 2**, the X-axis shows the expression level of hypoxia-inducible factor-1*α* (HIF-1*α*), which plays the role of an ‘angiogenic switch’ in the hypoxia microenvironment in many types of tumors, including IGC (43). The Y-axis shows the expression level of Cyclooxygenase-2 (COX2), which plays a crucial role in cancer development and clinical metastasis (44). We can also choose two other genes (such as EGFR and VEGF), as shown in the **Supplementary Material**. Here, there are still three stable states. There are some changes in the landscape topography, as different genes have different values for the three stable points.

On the IGC landscape, there are three stable state attractors, which are the normal, AG, and cancer (IGC) states, respectively. The definition of these three stable states is based on the biological functions and gene expression levels of the 17 genes in the gene regulatory network. The parameters we set in our model depend on the simulated gene expression levels of the 17 genes, and are all in agreement with the trends seen in the clinical data at different cancer stages (different states). P53 and APC are tumor suppressor genes that have high expression levels in the normal state but low expression levels in cancer cells. The other 15 genes have high expression levels in cancer and low expression levels in normal cells. The simulated gene expression levels of the 17 genes are all consistent with the experimental literature (details can be seen in **Supplementary Material Table S3**).

In **Figure 2**, the genes HIF-1*α* and COX2 have high expression levels in the cancer state, low expression levels in the normal state, and intermediate expression levels in the intermediate state (AG state) (45). The barrier height between the normal and AG states is relatively low, which indicates that AG infection and recovery is relatively achievable. The barrier height between AG and gastric cancer states is much higher, which indicates that gastric cancer formation and recovery is much more difficult. The barrier height can quantify the difficulty of attractor transfer from one state to another. The barrier height from gastric cancer to the AG state is very high, which shows why gastric cancer is so difficult to cure (or reverse).

To describe the gastric cancer progression process quantitatively, we quantified the dominant paths from normal to AG to gastric cancer states using previously-explored approaches (path integral approaches) (46, 47). In **Figure 2B**, we can see the dominant paths colored in red, blue, yellow, and purple, respectively, as the dominant paths from the normal to the AG state, from the AG to the normal state, from the AG to the gastric cancer state, and from the gastric cancer to the AG state, respectively. These dominant paths are separated and irreversible (48) as the rotational flux force (as part of the driving force) in addition to the gradient force makes the dominant path separate from the gradient direction of the potential. In our model, the driving force can be mathematically decomposed into two directions, the rotational flux force and the gradient force of the potential landscape. The green arrows represent the rotational flux force and the white arrows represent the gradient force direction. The dominant paths through the normal state to the AG and gastric cancer states are irreversible, which can help us understand why the processes of IGC formation and IGC treatment are separate and irreversible biological processes.

### 3.2 Simulations of the Effect of *H. pylori* Infection on IGC

To investigate the influence of *H. pylori* on IGC, we performed simulations to observe the cancer progression on the landscape. We used a term in the ODEs to simulate different degrees of *H. pylori* infection. The term *F*(*x_i_*) can be rewritten as *F*′(*x_i_*) = *F*(*x_i_*) + *H_i_* (*i* = 1,2…,17). The term *H_i_* is used to denote the degree of Helicobacter pylori infection on the related gene expression level. The value of *H* is set according to the experiments. If the gene expression level is increased from the *H. pylori* infection, the value of *H* will be >0. In the opposite case, *H* < 0.

**Figure 3:**
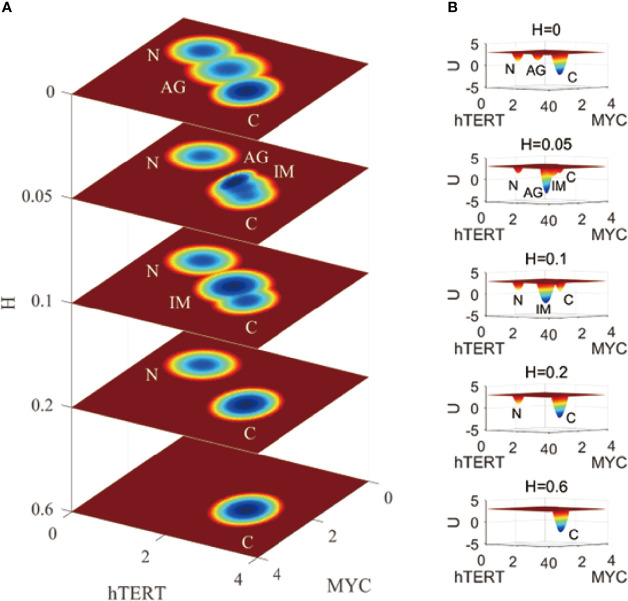
A comparison of the landscape topography variations fir IGC with Helicobacter pylori infection. The X and Y-axes represent the gene expression levels of hTERT and MYC, respectively, while the Z-axis represents the variations in H. H denotes the degree of Helicobacter pylori infection. N, AG, IM, and C represent the normal, atrophic gastritis, intestinal metaplasia, and gastric cancer states, respectively. **(A)** is the comparison of the 2-dimensional landscape. **(B)** is the comparision of the 3-dimensional landscape.

For **Figure 3**, we chose genes hTERT and MYC to show the landscape layers with variations associated with the development of IGC under the effects of *H. pylori* infection. *H. pylori* infection can result in the gene expression levels of both genes being increased. From **Figure 3**, we can see that when the term *H* = 0, the normal, AG, and gastric cancer states are visible on the first layer of the landscape. A value of *H* = 0.05 indicates infection. There are normal, AG, IM, and gastric cancer states on the second layer of the landscape. The probability of the AG state is dominant, indicating that the *H. pylori* infection accelerates the development of AG. When the term *H* = 0.1, there are normal, IM, and gastric cancer states on the third layer of the landscape. The AG state has disappeared and the IM state is dominant, which indicates that the *H. pylori* infection worsens gastritis and causes further change into intestinal metaplasia. When the term *H* = 0.2, there are normal and gastric cancer states on the fourth layer of the landscape. The IM and gastric cancer states gradually converge and merge into one. The cancer state becomes the dominant state. When the term *H* = 0.6, there is only one gastric cancer state on the fifth layer of the landscape. The normal state disappears and the cancer state is dominant. It is impossible for a patient to recover to their normal state while suffering from *H. pylori* infection can lead to the aggravation of AG, and then the appearance of the IM state. The IM state can be considered very close to the cancerous state during IGC development. When the *H. pylori* infection becomes more and more serious, the AG state disappears and the IM state becomes dominant, finally leaving only one gastric cancer state.

This simulation is on the epigenetic level to illustrate the progression and development of IGC when one gets infected with *H. pylori*. The regulations of the network do not change with this series of variations in the landscape. The effect of *H. pylori* infection results in variations in the IGC landscape. Depending on the degree of *H. pylori* infection, which is becoming more and more serious, landscape development is moving on the cancer direction. As AG and IM are seen in the development of the landscape, which demonstrates the dynamic process of IGC development as the degree of *H. pylori* infection changes.

### 3.3 Identifying Key Regulations of IGC Through Global Sensitivity Analysis

To further investigate the key regulations crucial to IGC therapy or prevention, we apply a global sensitivity analysis method to the landscape model. Each gene regulation or protein concentration can contribute to system dynamics. A small change in the regulatory strength of one gene in the gene regulatory network can lead to the whole landscape topography varying accordingly. As the barrier heights between the biological states can quantify the difficulty associated with transferring between the states, we calculate the variation in the barrier height when regulation strength is changed. The greater the variation, the more sensitive the regulation is.

**Figure 4:**
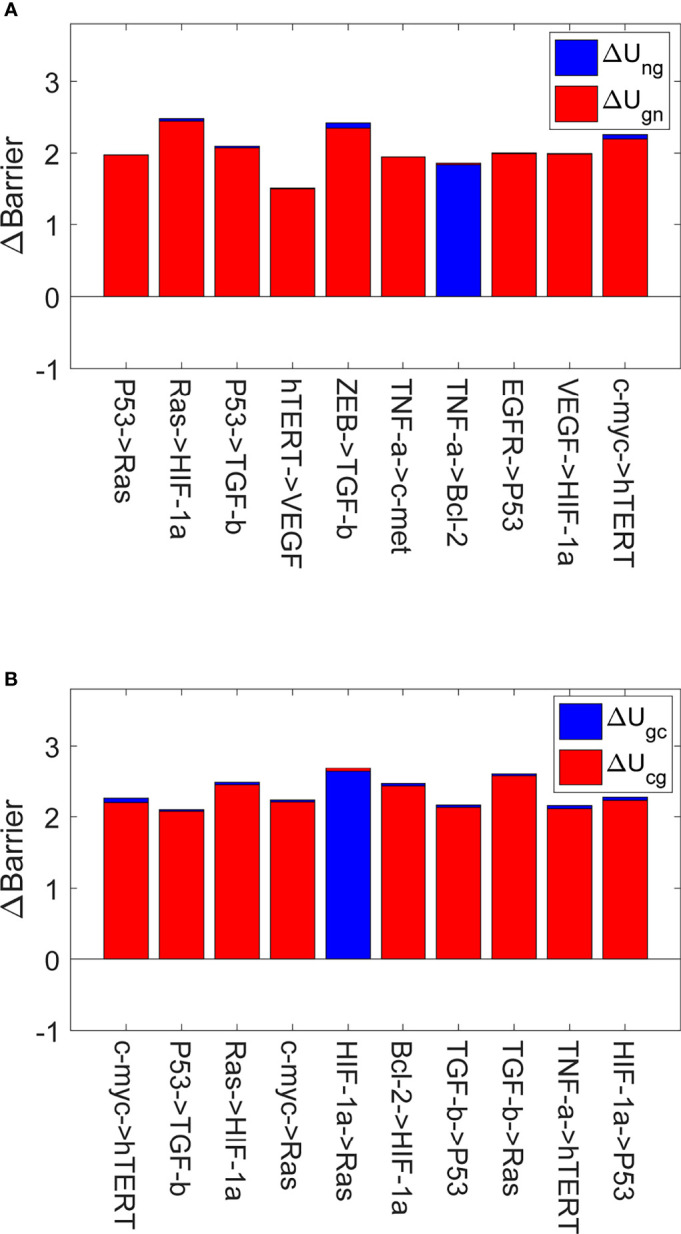
The global sensitivity analysis for IGC. The X-axis represents the top 10 most sensitive regulations, and the Y-axis represents variations in the barrier height (∆Barrier). **(A)** The variations in the barrier height between the normal and AG states. ∆Ung is the variation in the barrier height between the normal and AG states. ∆Ugn is the variation in the barrier height between the AG and normal states. **(B)** The variations in the barrier height between the AG and cancer states. ∆Ugc is the variation in the barrier height between the AG and cancer states. ∆Ucg is the variation in the barrier height between the cancer and AG states.

**Figure 4** shows the global sensitivity analysis for IGC. We selected the top 10 most sensitive regulations, which are shown in **Figure 4A**. When we changed the regulation strength to 0.9 of the original regulation strength, these regulations showed the most significant variations in Δ*U_ng_* and Δ*U_gn_*. Δ*U_ng_* is the variation of the barrier height from the normal state to the AG state. Δ*U_gn_* is the variation of the barrier height from the AG state to the normal state. From **Figure 4A**, we can see that the values of Δ*U_ng_* changed most significantly are for regulating RAS → HIF-1*α* and ZEB → TGF-*β*. The higher value of Δ*U_ng_* indicates that it is more difficult for the cells to transform from the normal state to the AG state than before. This is because the barrier height between the normal and the AG state is much higher than before. The variations of Δ*U_ng_* are most significant when the regulation strengths of the RAS → HIF-1*α* and ZEB → TGF-*β* are varied. This type of variation can be used in gastritis prevention as the cell transformation to the AG state becomes more difficult. When the regulation strength is reduced to 0.9 of the original value, the activation of the genes HIF-1*α* and TGF-*β* decreases and the concentrations of HIF-1*α* and TGF-*β* decrease accordingly. Experiments show that the expression levels of HIF-1*α* in gastric cancer patients are higher than those in healthy subjects (25). HIF-1*α* participates in the activation of numerous target genes to adapt to the hypoxic environment (49), which leads to gastric cancer development. We changed the regulation strength, which can cause the concentration of HIF-1*α* to decrease. This will inhibit the transcription of those target genes and reduce the ability of the cells to adapt to the hypoxic environment, which leads to higher Δ*U_ng_*. Therefore, reducing the regulation strength to 0.9 times the original regulation strength can inhibit the cells from adapting to the hypoxic environment, which can prevent the gastritis cells from developing into gastric cancer. Studies show that inhibiting TGF-*β* expression can serve as a potential therapeutic target or a biomarker for gastric cancer treatment (50, 51). Therefore, the decreased regulation strength can reduce the transcription of TGF-*β*, which increases in Δ*U_ng_* and inhibits gastritis from developing into gastric cancer.

**Figure 4B** shows the top 10 most sensitive regulations when we changed the regulation strengths to 0.9 times their original values. These regulations show the most significant variations of Δ*U_gc_* and Δ*U_cg_*. A higher value of Δ*U_gc_* compared to before indicates that switching from the AG state to the gastric cancer state becomes more difficult, because the barrier height between the AG and gastric cancer states is much higher. Reducing the regulation strength can inhibit the expression level of RAS, which increases in Δ*U_gc_* and helps inhibit gastric cancer progression and development. Such variation can be used in gastric cancer prevention, as the transformation of the cell from the AG state to the gastric cancer state becomes more difficult. When the regulation strength is reduced to 0.9 times the original value, the activation of the RAS gene decreases and the concentration of RAS also decreases accordingly. There are many studies showing that K-ras is associated with the development and progression of gastric cancer. Overexpression of K-ras can increase the risk of gastric cancer development (52) (K-ras is a protein of the RAS family). Reducing the regulation strength can inhibit the expression level of RAS, which increases in Δ*U_gc_* and helps inhibit gastric cancer progression and development.

**Figure 4** displays the most sensitive regulations on global topography in terms of the barrier height between normal and cancer states. When the regulation strengths of P53 → RAS, c-myc→ RAS, HIF-1*α* → RAS and TGF-*β* → RAS are reduced to 0.9 of the original values, the expression of RAS will decrease accordingly. Studies show that K-ras regulates cell survival, motility, proliferation, angiogenesis, and metastasis (53). Therefore, this participates in gastritis and gastric cancer formation and metastasis. When the regulation strengths of RAS → HIF-1*α*, VEGF → HIF-1*α*, and Bcl-2 → HIF-1*α* are reduced to 0.9 of the original values, the expression of HIF-1α is reduced accordingly. The gene HIF-1*α* can activate the transcription of many target genes to adapt to the hypoxic environment of cancer cells (54). The overexpression of the gene HIF-1*α* can induce cancer cell development. When the regulation strengths ofP53 → TGF-*β* and ZEB → TGF-*β* are reduced to 0.9 of the original values, TGF-β expression decreases. TGF-*β* promotes cancer-related characteristics in most gastric cancer cell lines (55). In TGF-*β* and HIF-1*α* gene expressions appear twice as that of the target genes in **Figure 4A**. Ras expression appears thrice in **Figure 4**, and the expressions of HIF-1*α*, P53, and hTERT genes appear twice as that of the target genes. We can pay more attention to these genes in designing strategies in clinical experiments or trials to prevent gastritis or gastric cancer formation.

### 3.4 Identifying Key Regulations of IGC With *H. pylori* Infection

In **Figure 3**, we can see that when a patient is infected with *H. pylori* (term *H* = 0.05), four stable states emerge, which are the normal, AG, IM, and gastric cancer states. To figure out which regulations are more sensitive to IGC with *H. pylori* infection, we performed a global sensitivity analysis on this condition.

**Figure 5:**
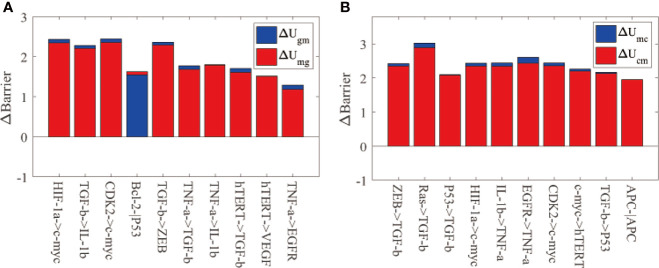
The global sensitivity analysis for IGC with Helicobacter pylori infection. The meanings of the X-axis and Y-axis are the same as **Figure 3**. **(A)** The barrier height variations between the AG and IM states. ∆Ugm represents the barrier height variation between the AG and IM states. ∆Umg is the barrier height variation between the IM and AG states. **(B)** The barrier height variation between the IM and cancer states. ∆Umc is the barrier height variation between the IM and cancer states. ∆Ucm is the barrier height variation between the cancer and IM states.

**Figure 5** shows global sensitivity analysis for IGC with *H. pylori* infection. We reduced the regulation strength to 0.9 of the original value. **Figure 5A** displays the top 10 regulations most sensitive to variations in Δ*U_gm_* and Δ*U_mg_*. Δ*U_gm_* is the variation in the barrier height from the AG state to the IM state. Δ*U_mg_* is the variation in the barrier height from the IM state to the AG state. The regulations for HIF-1α→c-myc and CDK2→c-myc cause the most significant changes in Δ*U_gm_*. The value of Δ*U_gm_* becoming higher indicates that it becomes more difficult for cells to transform from the AG state to the IM state as the barrier height is higher. This type of variation can be used to prevent cell transformation from the AG state to the IM state. As the activation strengths of HIF-1*α*→c-myc and CDK2→c-myc are decreased, the expression of c-myc will be reduced accordingly. Gene c-myc has been studied as a biomarker with which to identify *H. pylori* infection (56). Gastric cancer treatment and gastric cancer progression are complicated by aberrant expressions of c-myc (57). Therefore, inhibiting the expression of c-myc will benefit the treatment of IGC with *H. pylori* infection and prevent transformations from AG to an IM state.

**Figure 5B** displays the top 10 regulations most sensitive to variations in Δ*U_mc_* and Δ*U_cm_*. Δ*U_mc_* is the variation of the barrier height from the IM state to the gastric cancer state. Δ*U_cm_* is the variation of the barrier height from the gastric cancer state to the IM state. The regulation RAS→ TGF-*β* caused the most significant changes in Δ*U_mc_*. The value of Δ*U_mc_* becoming higher indicates that it becomes more difficult for cells to transform from the IM state to the gastric cancer state because the barrier height between the two states is higher. As the activation strength of RAS→ TGF-*β* is decreased, the expression of TGF-*β* will be reduced accordingly. TGF-*β* can trigger epithelial–mesenchymal transition (EMT) markers, which are crucial for canceration and metastasis. *H. pylori* infection can induce TGF- *β* to trigger the EMT pathway. When *H. pylori* is eradicated, TGF- *β* is inhibited from triggering the EMT pathway (58). TGF- *β* is a key gene in gastric cancer prevention and treatment, which we have identified in this study. C-myc and TGF- *β* are vital for the treatment and prevention of IGC from *H. pylori* infection.

From **Figure 4** and **Figure 5** we can see that the key regulations are different depending on whether the *H. pylori* is infected or uninfected. When infected with *H. pylori*, another state (IM state) emerged on the landscape, which is different from the IGC landscape without *H. pylori* infection. The c-myc gene is essential as it appears in the two key regulations (HIF-1α→c-myc and CDK2→c-myc). The c-myc gene is a biomarker to identify *H. pylori* infection in clinical trials (56). We can take other regulations such as TGF-*β*→ IL-1*β* and TGF-*β*→ ZEB, which are more sensitive in clinical experiments. The gene TGF-*β* is vital for both *H. pylori* infected and uninfected as it is sensitive to the two conditions when the cell states switch from AG (or IM) state to cancer state. TGF-*β* plays a critical role in cancer metastasis (58). TGF-*β* appears thrice in the top 10 regulations in **Figure 5B**, while TNF-*α* and c-myc genes appear twice. We should take these genes into consideration in designing strategies in clinical experiments for preventing of gastric cancer with *H. pylori* infection.

## 4 Conclusions

In this work, we have studied the formation and development of IGC in a systematic and quantitative way. We have built a gene regulatory network for IGC. The genes and gene regulations were collected through experimental literature research. The gene regulatory network reflects both genetic and epigenetic level information. After the construction of the gene regulatory network, we used ODEs to describe the dynamics of IGC. We then obtained a systematic landscape for IGC. There are normal, AG, and gastric cancer states on the IGC landscape. The landscape can provide us with a global overview of IGC progression and development, which can help us understand IGC formation systematically. The dominant paths can describe the IGC progression and dynamical transitions can help us understand the IGC development quantitatively. The dominant paths between neighboring states (the normal and AG states or the AG and gastric cancer states) are separate and irreversible. The irreversibility of the dominant paths explains why the IGC formation and recovery processes are complex and independent.

To investigate the effect of *H. pylori* infection on IGC formation, we simulated different degrees of *H. pylori* infection, resulting in variations in the landscape topography. When one is infected with *H. pylori* (H = 0.05), a state called intestinal metaplasia IM appears in the landscape, and the atrophic gastritis (AG) state becomes dominant. When the degree of *H. pylori* infection becomes serious, the AG state disappears and the IM state becomes dominant. As the degree of *H. pylori* infection increases, the normal state disappears, eventually leaving only one gastric cancer state. This demonstrates that *H. pylori* infection leads to gastric cancer progression and illustrates how *H. pylori* infection can increase the risk of gastric cancer development.

To further highlight the key regulations associated with IGC therapy and treatment, we performed a global sensitivity analysis and found three key regulations to be more sensitive than the others as the landscape topography varies. The three regulations are RAS → HIF-1*α*, ZEB → TGF-*β*, and HIF-1*α*→ RAS. We predicted that these regulations would serve as a guide for developing network-based anti-cancer drug targets.

This study provides a new approach and a novel yet simple model to analyze IGC in a global and systematic way. This model can help us understand the formation and development of IGC, not only from genetic variations but also from epigenetic modifications. Furthermore, *H. pylori* infection can be simulated and investigated with the landscape model. Global sensitivity analysis can help us determine which regulations are more sensitive for gastric cancer prevention or therapy. The results can help us develop clinical strategies by designing polygenic drugs to fight cancer.

## 5 Support Materials

### 5.1 Landscape-Flux Decomposition of the Driving Force

A gene regulatory network consists of genes and gene regulatory relationships, represented by nodes and edges in the network, respectively. We use an *n*-component vector ***x*** = (*x*_1_, *x*_2_,…, *x_n_*) to quantify the genes in the network. Here *n* is the number of genes in the network and *x_i_* (*i* =1,2,…, *n*) denote the expression levels (or protein concentrations) of the corresponding genes. A system of ordinary differential equations, written in the compact form $\dot{x}=F(x)$ , can be employed to study the deterministic dynamics of the network, where ***F*** (***x***) denotes the driving force of the deterministic dynamics.

In biological systems, stochastic fluctuations of internal or external origins are ubiquitous and may have a significant impact on the dynamics of the system. To incorporate the effects of stochastic fluctuations, a stochastic force *ξ*(*t*) may be attached to the ordinary differential equations $\dot{x}=F(x)$ . This leads to a stochastic differential equation of the form $\dot{x}=F(x)+\xi (t)$ , also known as the Langevin equation. The stochastic force *ξ*(*t*) modeling random fluctuations is assumed to be Gaussian white noise in time, with the mean〈 *ξ*(*t*) 〉=0 and the correlation 〈 *ξ*(*t*)*ξ* ^*T*^(*t*') 〉=2*Dδ*(*t*−*t*'). Here ***D*** is the diffusion matrix characterizing the fluctuation strength.

An equivalent description of the Langevin dynamics is in terms of the probability distribution *P*(***x***, *t*), whose time evolution is governed by the corresponding Fokker-Planck equation: ∂*P*/∂*t* = *-*∇ · [***F*** *P* - ∇· (***D*** *P*)]. It can also be written as ∂*P*/∂*t* = - ∇ · ***J***, with ***J*** denoting the probability flux. The steady state characterized by ∂*P_ss_*/∂*t* = ∇ · ***J*** *_ss_* = 0 is of particular interest. In an equilibrium system, the probability flux at the steady state vanishes, i.e. ***J*** *_ss_* = 0. In a non-equilibrium system, there is in general a nonvanishing probability flux at the steady state, i.e. ***J*** *_ss_* ≠ 0, which signifies the time-irreversible nature of the non-equilibrium steady state. From the expression ***J*** *_ss_* = ***F*** *P_ss_* – ***D*** · ∇*P_ss_*, the driving force ***F*** can be written in the landscape-flux decomposition form (59): ***F*** = -***D*** · ∇*U* + ***J*** *_ss_*/*P_ss_*. Here –***D*** · ∇*U* is the part of the driving force contributed by the gradient of the potential landscape *U* = -ln *P_ss_*, and ***J*** *_ss_*/*P_ss_* is the other part contributed by the probability flux that is associated with the nonequilibrium nature of the system.

### 5.2 Self-Consistent Mean Field Approach

Self-consistent mean field (60) serves as an effective approximation method of solving Fokker-Planck equations with a large number of variables. In this approximation, the joint probability distribution of all the variables is substituted by the product of marginal probability distributions of each variable, namely, *P*(*x*_1_, *x*_2_,…, *x_n_*, *t*) ~ Π*_i_ P* (*x_i_*, *t*), so that the latter can be solved in a self-consistent manner. The dimensionality of the problem is reduced significantly from *m^n^* to *m* × *n*, where *m* is the number of possible values each variable may take. This makes the computations much more feasible.

A further approximation is invoked to simplify the problem, which postulates *P*(*x*_1_,*x*_2_,…,*x_n_*, *t*) with the form of a multivariate Gaussian distribution. When the magnitude of the diffusion matrix ***D*** is small, the equations governing the mean vector $\bar{x}(t)$ and the covariance matrix ***σ*** (*t*) of the Gaussian distribution are given by:


$$\dot{x}(t)=F(\bar{x}(t))\;(3)$$

$$\dot{\sigma }(t)=A(t)\sigma (t)+\sigma (t)A^{T}(t)+2D.\;(4)$$

Here the matrix ***A*** has elements $A_{ij}(t)=\frac{\partial F_{i}(\bar{x}(t))}{\partial {\bar{x}}_{j}(t)}$ . Given the self-consistent mean field approximation, only the diagonal elements of *σ*(*t*) need to be considered. The combination of the self-consistent mean field approach and the Gaussian distribution approximation leads to the following form of probability distribution evolution for each *x_i_*:


$$P(x_{i},t)=\frac{1}{\sqrt{2\pi \sigma_{i}(t)}}\text{exp }\left\{-\frac{{\left[x_{i}-{\bar{x}}_{i}(t)\right]}^{2}}{2\sigma_{i}(t)}\right\}.\;(5)$$

The steady-state probability distribution of a monostable system with one fixed point can be easily obtained on the basis of Eq.(5) as a single Gaussian distribution. In a multistable system with more than one fixed point, the steady-state distribution may be constructed as a combination of multiple Gaussian distributions with the form *P_ss_* (***x***) = *Σ_k_ω_k_P_k_* (***x***), where *k* labels different fixed points, *ω_k_* represents the weight of each fixed point, and *P_k_* (***x***) is the Gaussian distribution corresponding to each fixed point.

### 5.3 The Path Integral Approach

Based on the Onsager-Machlup functional, the transition probability of the Fokker-Planck equation has the following path-integral formulation (46):


$$\begin{array}{c} P(x_{f},t_{f};x_{0},t_{0})=\int D\;[x(t)]exp\{-S[x(t)]\}\\ =\int D\;[x(t)]exp\left\{-\int L(x(t))dt\right\}. \end{array}\;(6)$$

In the above, ***x***_0_ denotes the initial state at time *t*_0_ and ***x*** *_f_* represents final state at time *t_f_*. *P*(***x*** *_f_*, *t_f_*; ***x***_0_, *t*_0_) is the transition probability from the initial state to the final state. The notation ∫ *D* [***x*** (*t*)] represents an integral over the all possible paths starting from the initial state ***x***_0_ at time *t*_0_ and ending at the final state ***x*** *_f_* at time *t_f_*. *L*(***x*** (*t*)) is the Lagrangian with the expression $L(x(t))=\frac{1}{4}(\dot{x}-F(x))\cdot D^{-1}\cdot (\dot{x}-F(x))+\frac{1}{2}\nabla \cdot F(x)$ . Its time integration gives the action *S*[***x*** (*t*)] = ∫ *L*(***x*** (*t*))*dt* associated with each path as in classical mechanics. The action *S*[***x*** (*t*)] determines the probability weight *e* ^-^ *^S^* ^[^ ***^x^*** ^(^ *^t^* ^)]^ contributed by the corresponding path. The summation (or integration) of these probability weights over all the paths gives the transition probability. Since the contribution of each path has the exponential form *e* ^-^ *^S^* ^[^ ***^x^*** ^(^ *^t^* ^)]^, the dominant path with maximum probability is the path with minimum action, which can be determined by the variational principle *δS*[***x*** (*t*)] = 0 and the resulting Euler-Lagrange equation. For non-equilibrium systems the existence of nonvanishing probability flux ***J*** *_ss_* cannot be ignored. As a consequence, the dominant kinetic paths in non-equilibrium systems are separated and irreversible (46).

## Data Availability Statement

The raw data supporting the conclusions of this article will be made available by the authors, without undue reservation.

## Author Contributions

Conception and design: CY and JW. Development of methodology: CY and JW. Acquisition of data: CY. Analysis and interpretation of data: CY and JW. Writing the manuscript: CY and JW. Study supervision: JW. All authors listed have made a substantial, direct, and intellectual contribution to the work and approved it for publication.

## Conflict of Interest

The authors declare that the research was conducted in the absence of any commercial or financial relationships that could be construed as a potential conflict of interest.

## Publisher’s Note

All claims expressed in this article are solely those of the authors and do not necessarily represent those of their affiliated organizations, or those of the publisher, the editors and the reviewers. Any product that may be evaluated in this article, or claim that may be made by its manufacturer, is not guaranteed or endorsed by the publisher.
